# Quantifying leaf herbivory: A guide to methodological trade‐offs and best practices

**DOI:** 10.1002/ecy.70308

**Published:** 2026-02-03

**Authors:** Tatiana Cornelissen, Gisele M. Mendes, Fernando A. O. Silveira, Wesley Dáttilo, Roger Guevara, Ramiro Aguilar, Maria Gabriela Boaventura, Ricardo Campos, Ek del Val, Guilherme Ramos Demetrio, Marcilio Fagundes, Rafael de Paiva Farias, Geraldo W. Fernandes, Tiago Fernandes, Inácio Gomes, Thiago Kloss, Juliana Kuchenbecker, Leandro Maracahipes, Frederico Neves, Lucas Paolucci, Cássio Cardoso Pereira, Elenir Queiroz, Letícia Ramos, Sérvio P. Ribeiro, Gustavo Q. Romero, Carolina Oliveira, Jhonathan O. Silva, Tathiana Sobrinho, Ricardo Solar, Heraldo Vasconcelos, Gabriela Zorzal, William C. Wetzel

**Affiliations:** ^1^ Centro de Síntese Ecológica e Conservação, Instituto de Ciências Biológicas Universidade Federal de Minas Gerais (UFMG) Belo Horizonte Minas Gerais Brazil; ^2^ Red de Ecoetología, Instituto de Ecología AC Xalapa Veracruz Mexico; ^3^ Red de Biología Evolutiva, Instituto de Ecología AC Xalapa Veracruz Mexico; ^4^ Instituto Multidisciplinario de Biología Vegetal, Universidad Nacional de Cordoba Córdoba Argentina; ^5^ Departamento de Biologia Geral Universidade Federal de Viçosa (UFV) Viçosa Minas Gerais Brazil; ^6^ Instituto de Investigaciones en Ecosistemas y Sustentabilidad, Universidad Nacional Autónoma de México Morelia Michoacán México; ^7^ Laboratório de Ecologia Vegetal Universidade Federal de Alagoas, Campus Arapiraca, Unidade Educacional Penedo Penedo Alagoas Brazil; ^8^ Laboratório de Biologia da Conservação, Departamento de Biologia Geral Universidade Estadual de Montes Claros Montes Claros Minas Gerais Brazil; ^9^ Instituto de Biologia, Universidade Federal da Bahia Salvador Bahia Brazil; ^10^ Knowledge Center for Biodiversity, Instituto de Ciências Biológicas, Universidade Federal de Minas Gerais (UFMG) Belo Horizonte Minas Gerais Brazil; ^11^ Departamento de Ciências Biológicas Universidade Federal dos Vales do Jequitinhonha e Mucuri Diamantina Minas Gerais Brazil; ^12^ Laboratório de Ecologia de Insetos Instituto de Ciências Biológicas, Universidade Federal de Minas Gerais Belo Horizonte Minas Gerais Brazil; ^13^ Laboratório de Ecologia e Comportamento, Departamento de Biologia Geral Universidade Federal de Viçosa Viçosa Minas Gerais Brazil; ^14^ Laboratório de Ecologia Evolutiva e Biodiversidade Instituto de Ciências Biológicas, Universidade Federal de Minas Gerais Belo Horizonte Minas Gerais Brazil; ^15^ Yale School of the Environment, Yale University New Haven Connecticut USA; ^16^ Ecotrop, Departamento de Biologia Geral Universidade Federal de Viçosa (UFV) Viçosa Minas Gerais Brazil; ^17^ Laboratório de Ecologia do Adoecimento e Florestas NUPEB‐ICEB, Universidade Federal de Ouro Preto, Campus Morro do Cruzeiro Ouro Preto Minas Gerais Brazil; ^18^ Laboratório de Interações Multitróficas e Biodiversidade, Departamento de Biologia Animal Instituto de Biologia, Universidade Estadual de Campinas (UNICAMP) Campinas São Paulo Brazil; ^19^ Laboratório de Ecologia Básica e Aplicada Universidade Federal do Vale do São Francisco Senhor do Bonfim Bahia Brazil; ^20^ Laboratório de Sistemática e Ecologia de Insetos, Departamento de Ciências Agrárias e Biológicas Universidade Federal do Espírito Santo, Campus São Mateus São Mateus Espírito Santo Brazil; ^21^ Instituto de Biologia, Universidade Federal de Uberlândia Uberlândia Minas Gerais Brazil; ^22^ Land Resources & Environmental Sciences Montana State University Bozeman Montana USA

**Keywords:** digital methods, folivory, herbivory intensity, herbivory protocol, herbivory quantification, leaf damage

## Abstract

Leaf herbivory is a ubiquitous ecological interaction that varies significantly in intensity across species, habitats, and biogeographic regions. Although quantification of leaf damage is crucial for understanding many ecological processes, the accuracy and precision of various damage estimation methods used by researchers, including visual estimation, digital image analysis, and artificial intelligence, have not been evaluated and compared. We use a phylogenetically diverse group of tropical plants to compare the accuracy and precision of damage estimation methods and use the results to provide a guide to herbivory estimation that balances the advantages and disadvantages of each method. We found that visual estimation tended to overestimate herbivory levels compared to digital methods but was 15 times faster and improved in accuracy and speed with training. Conversely, deep‐learning algorithms underestimated herbivory relative to image analysis with ImageJ when it was on the margin, but showed similar accuracy for damage inside of leaf margins. Our results indicate that while visual methods allow for rapid assessment of large sample sizes and are suitable for detecting broad patterns of damage, image analysis is crucial for accurate and precise quantification. The disadvantages of each method, however, can be minimized through proper training and efficient use of each tool, and we therefore provide a guide of practical approaches to herbivory estimation.

## INTRODUCTION

Herbivory is pivotal in energy transfer from plants to higher trophic levels in terrestrial and aquatic ecosystems, across all biogeographic regions (Díaz et al., 2003; Emer et al., 2024; Futuyma & Agrawal, 2009; Price, 2002; Turcotte, Davies, et al., 2014; Turcotte, Thomsen, et al., 2014); yet, it also varies significantly in intensity at all scales (Wetzel et al., 2023) and across water and land plants. Macrophyte herbivory is widespread in the world's oceans (Bakker et al., 2016), in freshwater systems, and it is a key factor shaping aquatic ecosystems with an important role in biomass and energy transfer (Cébrian & Duarte, 2016). For terrestrial plants, research reveals that even minor levels of leaf damage by herbivores potentially impact fitness, reducing growth, reproduction, and competitive performance (Bigger & Marvier, 1998; González‐Browne et al., 2016). However, despite decades of research, accurately assessing the impacts of herbivory on plant community dynamics and ecosystem processes across habitats remains challenging (Janzen, 1981; Johnson et al., 2016; Louda, 1984). Recent efforts have aimed to quantify the extent of leaf removal of Angiosperms by insect herbivores on a global scale (see Kozlov et al., 2015a, 2015b; Liu et al., 2024; Mendes et al., 2021; The Herbivory Variability Network, 2023; Turcotte, Thomsen, et al., 2014), but herbivory estimation methods have varied, which hinders direct comparisons and syntheses of published data. Factors contributing to the lack of standardization include non‐random selection of host plants (e.g., focus on crops or commonly widespread species), imprecise quantification of the amount of leaf damage (Johnson et al., 2016; Kozlov et al., 2014), and the application of novel methods tested on a limited set of species (Vieira et al., 2025; Wang et al., 2024).

**FIGURE 1:**
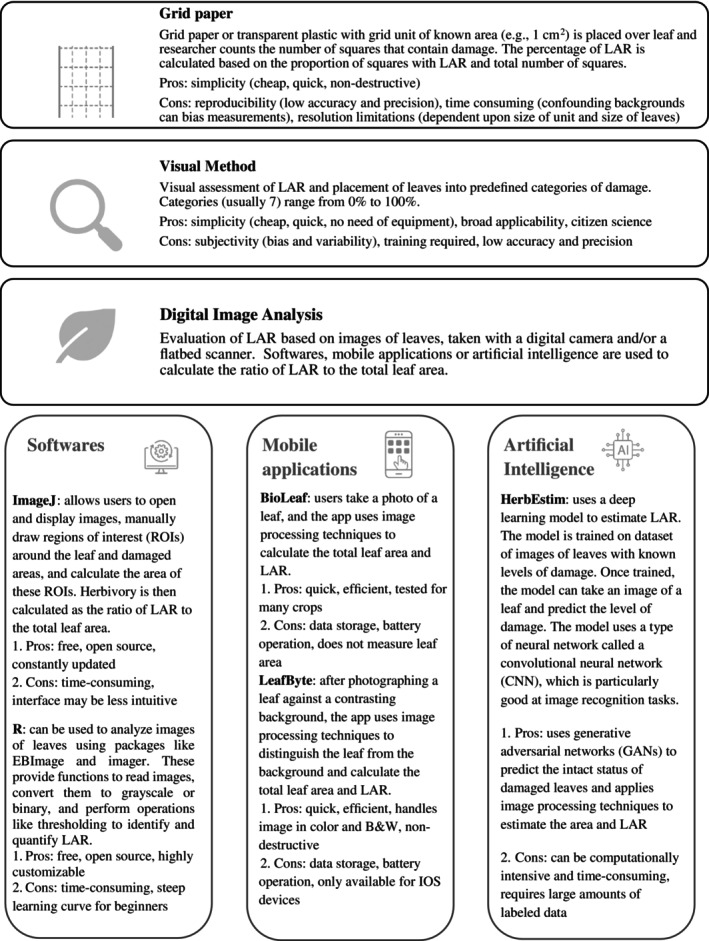
Methods used to quantify herbivory by insects available in the scientific literature. Methods are divided into three main categories, and tools available for image‐based methods are further explained. We provide pros and cons for each method based on simplicity (ease of use) and reproducibility (precision and accuracy). LAR, leaf area removed. Figure created by Tatiana Cornelissen using icons from Flaticon (www.flaticon.com) under Flaticon Basic License.

Given the ubiquity of herbivory across different ecosystems and plant lineages, numerous methods have been proposed to estimate leaf consumption by herbivores (Figure 1). For example, visual estimates of herbivory, in which the extent of leaf removal is assessed with unaided eyes, have a rich historical background. Visual estimation (e.g., Alliende, 1989; Dirzo & Domínguez, 1995; Lincoln & Mooney, 1984) continues to be widely employed due to its simplicity and minimal time investment. Visual estimates can be quickly performed in the field by people without specialized equipment, making it accessible to researchers anywhere (Johnson et al., 2016). This approach enhances our understanding of global patterns of herbivory and its variation between and within plant species (Xirocostas et al., 2022). The visual method can take two forms: simple estimation—in which damage is quantified as the estimated percentage of leaf area removed—or categorical estimation, in which damage falls into predefined classes (e.g., 12%–25%, 26%–50%, 51%–75% of leaf area lost) (see Dirzo & Domínguez, 1995). Following categorization, the leaf damage index (LDI) is calculated as the sum of leaf frequencies in each category, multiplied by the median value of the respective category, divided by the total number of collected leaves (see Kozlov et al., 2014; Kozlov & Zvereva, 2017).

Despite its simplicity and effectiveness, visual estimates of herbivory have inherent limitations. Because they are based on the human eye, the repeatability and precision of these measurements have been questioned and rarely validated (Johnson et al., 2016). As a solution, some researchers favor broad categories (such as those proposed by Alliende, 1989; Dirzo & Domínguez, 1995), while others suggest using finer gradations (Kozlov & Zvereva, 2017). However, a wide range of uncertainty persists even with narrower categories of leaf damage, with leaves with very different amounts of area removed falling into the same damage classes. Given that even low levels of herbivory—below 5% of leaf tissue removal—can significantly impact plant performance (Marquis, 1984), the breadth of the categories used in the LDI can lead to biased estimates of the actual herbivory levels, introducing confounding factors to the impacts of leaf removal on plant fitness. To account for some of these issues, a free application, The Zax Herbivory Trainer (Xirocostas et al., 2022), was released in 2022 to support training researchers to minimize biases on herbivory estimation, especially when dealing with multiple leaf forms, under field conditions, and with time constraints. The Zax Herbivory Trainer is an online app and has been used by 1600 users as of August 2024, indicating that the visual estimate of herbivory is a valuable resource.

Alternatively, image‐based methods have emerged in an attempt to increase the precision and accuracy in estimating leaf damage (Figure 1). One such method involves digitizing leaves using a flatbed scanner or digital camera, followed by image processing to quantify the extent of tissue damage. This procedure is often conducted using Adobe Photoshop (adobe.com), Scion (https://www.scionresearch.com/), or ImageJ (https://imagej.net/ij/) software. Over the past 25 years, ImageJ has played a pivotal role in advancing herbivory quantification across plant organs and plant species due to its user‐friendly interface and cross‐platform compatibility (Abràmoff et al., 2004; Schneider et al., 2012). More recently, coding and the development of packages have enabled using digital images to estimate leaf herbivory within R (*EBImage* by Pau et al., 2010; *LeafArea* by Katabuchi, 2015; *pliman* by Olivoto, 2022). These packages automate the measurements of leaf area and area removal, allowing for the analysis of larger sets of leaves into a single file, potentially reducing the time required for measurements. In addition to these digital tools, mobile applications like BioLeaf (Machado et al., 2016) and LeafByte (Getman‐Pickering et al., 2020) were developed and released over the last 5 years. More recently, deep‐learning and machine‐automated methods (e.g., HerbiEstim by Wang et al., 2024) were developed as alternative methods to measure herbivory on large datasets, usually within seconds (Vieira et al., 2025; Wang et al., 2024).

Contrary to visual estimates of herbivory, methods based on digital image analysis require a series of steps: collecting, processing (e.g., drying, pressing, and scaling), and image acquisition (e.g., digitizing leaves or camera use) before measurements can be taken. These methods are time‐consuming, but they are expected to yield precise and reliable estimates of leaf damage, facilitating comparisons among different plant individuals, populations, species, and sites. Therefore, a trade‐off between precision and time needs to be optimized. Despite the use of these methods (Johnson et al., 2016; Ullah et al., 2020), there are still gaps in our understanding of how methods, leaf traits, or image processing influence the accuracy and precision of herbivory estimates, especially for wild species in natural ecosystems.

Prior studies assessing the efficacy of visual estimates and digital tools for quantifying herbivory have primarily focused on single or a few plant species or crops. Therefore, our research uses an extensive herbivory dataset from tropical plants to provide a field guide for herbivory estimation that considers and balances advantages and shortfalls of different approaches. The aims of our study were to (1) evaluate the accuracy of different methods for measuring herbivory across a large dataset comprising phylogenetically diverse plants with varying levels of natural and artificial leaf damage and (2) provide a handbook and methodological guidelines that allow repeatability and comparability across studies. To achieve these goals, we experimentally evaluated the accuracy of different herbivory quantification methods at the leaf level and provide recommendations to support quantitative estimates of herbivory considering accuracy, precision, and time spent on measurements.

## METHODS

### Database

This study is part of the TropHerb Network, an inclusive, global network of researchers focused on studying herbivory and florivory in Neotropical plants. From a database (Mendes et al., 2021) encompassing over 50,000 leaves of 209 species sampled between 2017 and 2023, the core group of TropHerb Network selected different sets of leaves and different plant species (Appendix S1: Figure S1) to test our hypotheses, considering interspecific variability in leaf size, morphology, herbivory levels, and the availability of intact leaves to create artificial herbivory levels. Briefly, leaf samples were sampled from natural habitats across 40 sites spanning 20° latitudes in the tropics from March 2017 to February 2023 (see Mendes et al., 2021). Collaborators of TropHerb identified and tagged the five most abundant woody species in each site to ensure intraspecific variability. They collected 50 leaves randomly around the canopy from each plant, yielding 250 leaves per species. From this larger dataset, the TropHerb core group selected 79 different plant species from 35 different plant families and evaluated more than 2000 leaves in this study. Different sets of plants were used to answer different questions regarding methods to estimate herbivory and the effects of image quality on measurements.

### Quantification of herbivory through visual estimates

#### Natural herbivory estimates

The TropHerb core group shared a questionnaire among 18 researchers from the network, requesting them to visually estimate the percentage of leaf area consumed by herbivores using images from 24 different plant species. These species were chosen among the 79 plant species to represent a broad spectrum of leaf traits (shapes, sizes, colors, and types of margin) and a wide range of damage levels, ranging from 0% (intact leaves) to 70% (highly damaged leaves). The TropHerb core group randomly selected 30 leaves for herbivory estimates. These leaves had been previously measured for herbivory levels percentage of leaf area removed using ImageJ and EBImage (see details in Mendes et al., 2021). All leaves showed natural herbivory levels by insects onto the leaf lamina as holes or as eaten margins. After visual estimation of herbivory, measurements were compared to herbivory estimates of these same leaves using two software tools (ImageJ and EBImage on R) and the deep‐learning machine algorithm HerbiEstim (Wang et al., 2024). The TropHerb core group compared herbivory levels across all four methods using average herbivory per plant. We then calculated the accuracy of visual estimation of herbivory by computing the delta‐herbivory estimation, defined as the discrepancy between the average leaf area removed obtained through digital image analysis in ImageJ, EBImage, and HerbiEstim and the visually estimated measurement performed by researchers.

#### Artificial herbivory estimates

To investigate the effects of researcher training on herbivory quantification, the TropHerb core group selected 100 intact leaves from 20 plant species. Leaves were subjected to different levels of artificial herbivory using a paper punch with a known diameter of 7.1 mm^2^, and total leaf area was measured using ImageJ and EBImage on R. This was done to ensure that the precise amount of herbivory was accurately known. Each leaf received 1, 2, 3, 5, 7, 12, or 18 holes, distributed across the leaf lamina while avoiding the leaf margins, simulating herbivory by chewers. Leaves were individually digitized with a ruler for scale purposes only, and this set of leaf images was distributed among 17 researchers at the Institute of Ecology of Mexico (INECOL). All researchers had no prior knowledge of the aims of the project. Herbivory was visually estimated by each researcher after examining the images of each leaf and recording the percentage of leaf area removed and the time (in minutes) required to evaluate the set of 100 leaves. Researchers were then asked to complete the ZAX online training (Xirocostas et al., 2022) and re‐evaluate the same sample of leaves, visually estimating herbivory without referring to the previous measurements. We then calculated the delta‐herbivory estimation (difference between the visual estimate of herbivory and the average known amount of herbivory) before and after ZAX training.

### Comparisons between different methods to quantify herbivory

To evaluate herbivory accuracy among three commonly used methods—visual estimation, ImageJ, and EBImage on R—a total of 64 plant species from 31 families were evaluated. A set of 250 leaves was sampled for each species (50 leaves from five individuals of each species). From this set of 250 leaves, the TropHerb core group randomly sampled 30 leaves per plant species through a drawing process. If a selected leaf was intact, we conducted another draw until we obtained a set of 30 leaves with varying levels of damage by natural herbivory for each plant species. This procedure ensured that all 1920 leaves had some sign of herbivory. Additionally, we recorded the time taken (in minutes) to classify and measure each set of 30 leaves. We opted not to use mobile applications (e.g., LeafByte and BioLeaf) due to the specific requirements of operating systems, costs associated with applications, image storage, and because some of those applications do not estimate the total leaf area. We did not use HerbiEstim deep learning algorithm (Wang et al., 2024) for this particular set of leaves, as our results (see below) indicated that this method did not return measurements of leaf area removal for several images of leaves that experienced herbivory on leaf margins.

For visual estimation of herbivory, we classified each sampled leaf (*n* = 30 per plant species) into one of the following damage categories: 0: intact leaf; 1: any levels of damage up to 1% of the leaf area removed; 2: between 1.01% and 5% of the leaf area removed; 3: 5.01%–25%; 4: 25.01%–50%; 5: 50.01%–75%; and 6: over 75%. We then calculated the herbivory index (HI) as follows: we multiplied the number of leaves in each damage category by the average value of the damaged leaf area. The HI was then obtained using the equation: HI = Ʃ(*ni*) × *i/* *N*, where *ni* represents the number of leaves in the *i* damage category, *i* is the median value of damaged leaf area, and *N* is the total number of sampled leaves.

To quantify herbivory using ImageJ, we randomly sampled leaf images (*n* = 30 for each plant species) and calculated: (1) total leaf area and (2) total area lost due to herbivory. Each image underwent initial processing including calibration to millimeters, conversion of the image to black and white, determination of the leaf area, filling in the leaf contour when necessary, and checking for shadows or spots that could be mistaken for leaf area or removed leaf area (see Appendix S1: Section S1). After obtaining all measurements, we determined the level of herbivory as a percentage of leaf area lost.

For herbivory measurements using R Core Team software, we used the EBImage package (Oleś et al., 2024; Pau et al., 2010) and a custom code (Figshare doi: 10.6084/m9.figshare.27123372). The images were initially processed using GIMP 2020 software, and a protocol for image processing was developed and made available to all researchers. Similar to ImageJ, the processing involved calibration to mm, optional conversion of the image color to black and white, delimitation of the leaf area (threshold), filling in the leaf contour when necessary, and checking for shadows or spots that could be mistaken for leaf area or removed leaf area. Following processing, we analyzed the images using the EBImage package in the R Core Team software to estimate the percentage of leaf area removed by herbivores.

We analyzed each leaf in this dataset (*n* = 1920) using all three methods (visual estimation, ImageJ, and EBImage in R) by at least two researchers. We also evaluated the time (in minutes) required to analyze each leaf. For ImageJ and R, we additionally accounted for the time needed to digitize the leaves and process the images before measurements. We opted not to use deep‐learning methods for this dataset, as this tool did not recognize leaf images in 20% of our dataset (see results below), especially when damage was concentrated on leaf margins.

### Comparisons between image data and leaf traits in herbivory estimates

To assess the influence of image processing method and leaf traits on herbivory estimates and the time required for measurements, we selected 20 leaves per species (*n* = 1280 leaves from 64 plant species) representing a diverse range of image data, including image color (colored vs. black and white), image type (digital photograph vs. digitized image with the aid of a flatbed scanner), and number of leaves per image (individual leaf in a single image file vs. all leaves in a single image file). We also considered leaf traits such as size (small = up to 6 cm^2^ and large = greater than 60 cm^2^), surface color (light vs. dark), the digitized surface (abaxial or adaxial), leaf margins (smooth vs. irregular), and herbivory levels (low = less than 5% of leaf tissue removal, intermediate = 6%–15% leaf tissue removal, and high = above 25% of leaf tissue removal).

The images were processed in GIMP software following the standardized protocol outlined in Appendix S1: Section S1. For each original image containing the 20 leaves/species, we generated 42 additional images. This set of images included one color image with all 20 leaves, one black‐and‐white image with all 20 leaves, 20 individual color images (each containing one leaf from the original image), and 20 individual black‐and‐white images (each containing one leaf from the original image).

To evaluate the influence of the type of image acquisition method on herbivory measurements, we included digitally scanned images and photographs of the 20 leaves. We ensured a light background for dark leaves and vice versa and used flash to avoid shadows in the digital photograph. A NIKON camera (D3100) was mounted on a tripod with a fixed height (30 cm) and stable wooden base for capturing digital photographs to standardize image quality and pixel count on the scale. After processing, herbivory was calculated using only the EBImage package in R.

### Data analysis

To compare herbivory quantification among the four methods (visual estimation, ImageJ, EBImage in R, and HerbiEstim deep‐learning algorithm), we firstly built a phylogenetic generalized least square (PGLS) model, initially reconstructing the phylogenetic tree using *phytools* (Revell, 2012) and *nlme* package (Pinheiro et al., 2025) in R software using the mega phylogeny by Qian and Jin (2016). We included the phylogenetic distances and expected covariance under a Brownian motion model in the model's random part, and the different methods to detect herbivory levels were used as fixed factors. For this same dataset of 24 species, we calculated delta herbivory as the difference between visual estimates and estimates derived from image analysis. Because there was no effect of phylogenetic relatedness on herbivory detection, we assumed independence across plant species in the analyses. The difference in delta‐herbivory estimation of visual estimates before and after training researchers on ZAX was tested using a one‐tailed paired sample *t* test.

To assess the effects of methods, image data, and leaf traits on herbivory estimates, we first built a PGLS model using 64 plant species, and due to the lack of a phylogenetic signal on herbivory variation among all factors evaluated, we proceeded with generalized linear mixed‐effects models (GLMMs) with beta distribution, using herbivory level as the response variable and the method/tool as the predictor variables. To compare the time spent generating herbivory estimates, we used method, image data, and leaf traits as explanatory variables, with time in minutes as the response variable. We incorporated species identity as a random effect in all models as different species were used for different models. Whenever necessary, we performed contrast analyses post‐GLMMs to highlight differences between measurement groups (see complete statistical report in Appendix S1: Section S2). The analyses were run using the software R 4.1.1 (R Core Team, 2023).

## RESULTS

### Quantification of herbivory through visual estimates

**FIGURE 2:**
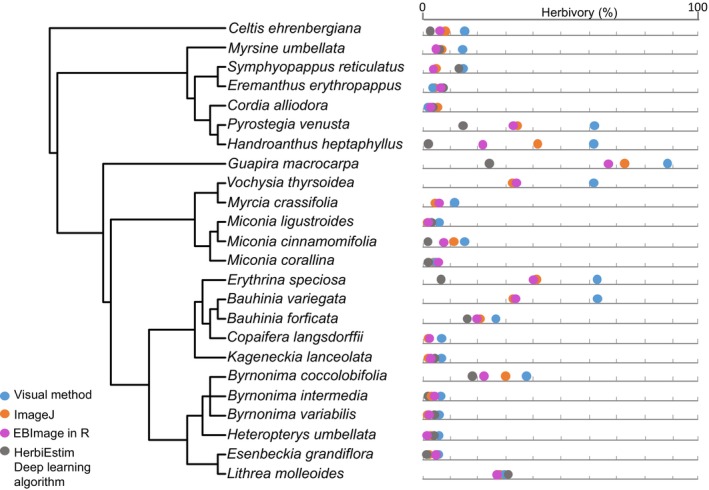
Phylogenetic tree and levels of herbivory in 24 plant species belonging to 14 botanical families. Different colored dots indicate each one of the four methods used to estimate or measure herbivory. In five plant species, the deep learning machine algorithm was not able to return measurements, hence the absence of gray dots.

**FIGURE 3:**
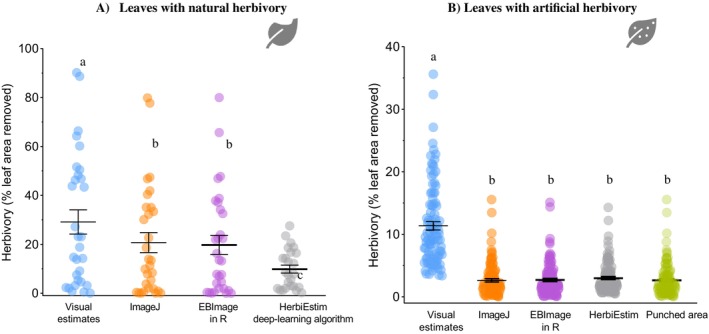
(A) Herbivory level (mean ± SE) on 30 leaves of 24 plant species as a function of the method used to estimate natural herbivory (Visual method: 29.15 ± 4.95; ImageJ: 20.72 ± 4.08; EBImage: 19.73 ± 3.85; HerbiEstim: 9.85 ± 1.61) and (B) Herbivory level (mean ± SE) of 100 leaves of 20 plant species with artificial herbivory as a function of the method used to estimate leaf area loss (Punched area: 3.54 ± 0.32; Visual method: 11.37 ± 0.65; ImageJ: 3.63 ± 0.26; EBImage: 3.49 ± 0.27; HerbiEstim: 3.00 ± 0.24). Figure created by Tatiana Cornelissen using icons of leaves from Microsoft PowerPoint.

Overall, the visual method consistently provided the highest estimates of herbivory. For leaves with natural herbivory caused by chewing insects, the visual method yielded higher estimates than image analysis (ꭓ^2^ = 38.34, df = 3, *p* < 0.0001) in 24 plant species (Figure 2). For these species, visually estimated herbivory was higher than image analysis in 87% of the species. Quantification of herbivory using ImageJ and EBImage yielded 20.7% (±22.4 SD) and 19.7% (±21.1 SD), respectively, of leaf area removed, whereas visual estimation yielded 29.1% (±27.1 SD) damage (Figure 3A), with a delta herbivory of about 9%. Deep‐learning machine algorithms yielded lower estimates of herbivory (9.8% ± 8.07 SD of leaf area removed), and in 20% of the species here evaluated, the algorithm could not read and interpret the leaf area and leaf damage from images. This was particularly common when leaves had damage on leaf margins instead of damage dispersed over the leaf blade.

**FIGURE 4:**
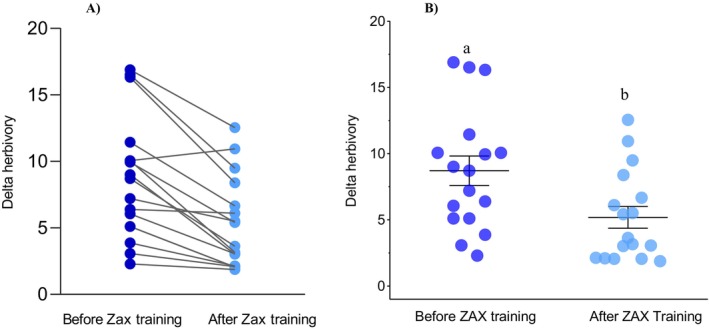
(A) Delta‐herbivory estimation (difference in herbivory level between visually estimated and known measurements of artificial herbivory of 100 leaves of 20 plant species) before and after training in ZAX platform. Each dot connected by a line indicates the average estimates of individual researchers (*n* = 17) and (B) delta‐herbivory estimation (difference in herbivory level between visually estimated and known measurements of artificial herbivory of 100 leaves of 20 plant species) as a function of researcher training in herbivory measurements using ZAX platform. Lines indicate mean ± SE. Significant differences at α = 0.05 are indicated by different letters.

For leaves with artificial herbivory, the visual estimation method overestimated herbivory by 5‐fold (ꭓ^2^ = 115.2, df = 3, *p* = 0.0002, Figure 3B, Appendix S1: Figure S2), with no significant difference in estimate among the other three methods (*p* = 0.90). We detected a positive effect of training on the ZAX platform on herbivory estimation (Figure 4), with a reduction of delta herbivory before and after training in 95% of the researchers (Figure 4A) and a reduction of 13 min (±4.45) in the time required to estimate herbivory. Delta herbivory for all plant species was significantly smaller after (5.18 ± 0.82) compared to estimates taken before (8.70 ± 1.11) ZAX training (*t* = 5.58, df = 16, *p* < 0.0001, Figure 4B).

**FIGURE 5:**
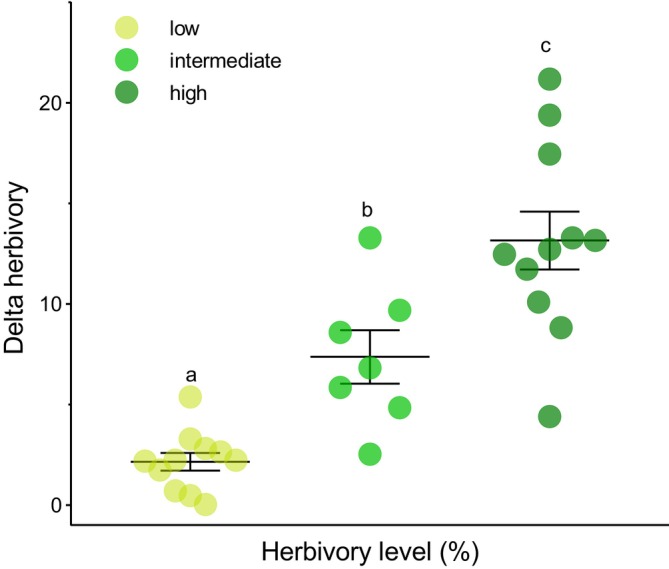
Difference between visually estimated herbivory levels versus those measured through image analysis (mean ± SE) as a function of herbivory level categorized as follows: Low = 0%–5% leaf tissue removal (*n* = 11 leaves); intermediate = 6%–15% leaf tissue removal (*n* = 7 leaves); and high = more than 20% leaf tissue removal (*n* = 11 leaves). Different letters indicate significant differences between herbivory levels, according to contrast analysis after generalized linear mixed‐effects model (GLMM). Each point represents a leaf.

Delta herbivory also varied according to the categories of leaf damage (*F*_2,27_ = 38.476, *p* < 0.0001, Figure 5). At low herbivory levels, delta herbivory was significantly lower (2.15 ± 0.44) compared to intermediate (7.37 ± 1.32) and high (13.16 ± 1.44) levels of leaf damage. We did not observe any effects of leaf size on the difference between visually estimated and digitally measured levels of herbivory (*F*_1,14_ = 1.276, *p* = 0.278).

### Comparisons between different methods to quantify herbivory

The quantification of herbivory in 1920 leaves of 64 plant species depended on the method used, with visual estimates significantly overestimating herbivory (ꭓ^2^ = 15.01, df = 2, *p* = 0.00055, Appendix S1: Figure S3), with no difference in herbivory accuracy using ImageJ or EBImage in R. Measurements of herbivory were 10 times quicker using visual estimates (3.8 ± 0.62 min), compared to ImageJ (41.03 ± 2.71 min) and EBImage, which took significantly longer (54.61 ± 3.82 min) (ꭓ^2^ = 106.478, df = 2, *p* = 0.0001) (Appendix S1: Figure S3).

### Comparisons between image data and leaf traits in herbivory estimates

Image color did not affect the accuracy of leaf herbivory measurements (ꭓ^2^ = 0.029, df = 1, *p* = 0.864) nor the time required for leaf measurements (ꭓ^2^ = 0.719, df = 1, *p* = 0.396) (Appendix S1: Figure S4). Similarly, there was no effect of the image digitization technique (scanned or photographed) on the accuracy of leaf herbivory measurements (*p* = 0.628) or the time required to perform these measurements (*p* = 0.450). The number of leaves per image did not affect the accuracy of leaf herbivory measurements (ꭓ^2^ = 0.010, df = 1, *p* = 0.920) (Appendix S1: Figure S4). Still, the use of a set of leaves significantly reduced the time to perform the measurements (ꭓ^2^ = 12.17, df = 1, *p* = 0.0004) compared to estimates on individual leaves (Appendix S1: Figure S4). We did not detect any effects of the leaf surface (adaxial or abaxial) used for measurements on the accuracy or time to estimate leaf herbivory (ꭓ^2^ = 1.35, df = 1, *p* = 0.245).

We found significant differences in the time required to estimate herbivory depending upon herbivory intensity, with leaves with lower herbivory levels requiring more time to be measured (ꭓ^2^ = 37.126, df = 1, *p* < 0.0001, Appendix S1: Figure S5). Additionally, herbivory on larger leaves required more time to be measured (Appendix S1: Figure S5). Still, there was no statistical difference in the time taken to estimate herbivory between leaves with different leaf face colors (light or dark) (ꭓ^2^ = 0.51, df = 1, *p* = 0.475) or different leaf margins (smooth or irregular) (ꭓ^2^ = 1.32, df = 1, *p* = 0.231).

## DISCUSSION

Our study revealed that visual estimates of herbivory were significantly faster but consistently higher than digital methods across a diverse range of distantly related plant species. This overestimation was particularly pronounced in leaves with artificial herbivory, where visual estimates exceeded the known damage by approximately five‐fold. This scenario, involving numerous small, scattered holes, appears to represent a worst‐case challenge for herbivory estimation, inflating error and the delta herbivory. While this level of discrepancy may not always occur with natural herbivory, it underscores the risk and potential magnitude of bias associated with the visual method. Conversely, deep‐learning machine algorithms yielded lower estimates of herbivory, with almost 60% of the assessed plants exhibiting values approximately 50% lower than the ones based on image analysis on software like ImageJ and R, which performed very similarly. Underestimation of herbivory by artificial intelligence was particularly pronounced in leaves with damage on the leaf margins, whereas the accuracy of herbivory levels was indistinguishable between methods in digitized leaves with artificial herbivory widespread across the leaf lamina. While visual estimates are a quick and widely used method (Frost, 2022; Xirocostas et al., 2022), they were influenced by factors such as the researcher's experience and training in herbivory measurements, the intensity of herbivory, and variations in leaf traits. Additionally, when herbivory is concentrated on leaf margins, as is common with caterpillar damage, reconstructing the total leaf area to estimate the leaf area eaten can be challenging. Inaccuracies may occur due to the need to reconstruct the original leaf area before estimating the area removed by herbivores. These issues can bias herbivory estimates regardless of the method used. However, we suggest that the visual method is more prone to errors than methods based on digital images, which can be processed to reconstruct leaf area. Although the visual method requires less time to estimate herbivory—allowing for a large number of samples to be quickly and easily assessed—we recommend that it should not be used without proper training. While visual estimation may be a poor choice for precise comparisons of herbivory within groups of plants with similar herbivory levels, it might be the only practical option for studies where the primary goal is to describe very large‐scale patterns or major differences across systems, and where the sampling scale makes digital methods logistically prohibitive. Indeed, our results suggest that in the time it takes to collect data from 30 leaves using digital methods, one could collect data from 450 leaves using visual estimation, allowing for considerably broader sampling. In such cases, it is imperative that all estimates within a study are made by the same observer, preferably one who has undergone training. We suggest that visual estimation can be used successfully, for example, as a first step in herbivory studies and as an aid in determining sampling sizes for more precise subsequent estimation with image analysis.

Our findings show that visual estimates were 3‐ to 5‐fold higher than measurements obtained using digital methods based on leaf images. This discrepancy was particularly high in severely damaged leaves, a typical pattern in tropical rainforests (Mendes et al., 2021). In such cases, the gap between visual estimates and digital estimates of herbivory levels significantly widened. The average 8% difference between visual and digital herbivory estimates using ImageJ and EBImage represents a significant discrepancy in the context of herbivory impacts because even relatively minor levels of leaf damage can adversely affect plant fitness, triggering premature leaf abscission, physical and chemical defenses, and imposing metabolic costs that can impact growth and reproduction (e.g., Kozlov & Zvereva, 2017). Consequently, visual estimation is less effective at quantifying minor differences in damage levels and is more suited to describing overall patterns and quantifying large differences between plants that are heavily damaged and those that are mostly free of damage.

Digital methods relying on leaf images and software, in contrast, provided a more accurate estimate of leaf damage but were more time consuming. Factors such as the number of leaves that can be included in a single image, leaf size, and leaf damage influenced the time required for measurements, but did not affect the accuracy of herbivory measurements. We also demonstrated that the online training in herbivory analysis was enough to reduce delta herbivory by more than 50% and is strongly encouraged to reduce herbivory estimation biases when employing the visual method (see Xirocostas et al., 2022).

Highly damaged leaves (approximately 20% of leaf area lost) exhibited the highest delta herbivory values. This indicates that researchers typically overestimate damage at higher levels of herbivory. We suggest that overestimating highly damaged leaves can be attributed to the individual's visual perception of herbivory. Because researchers must visually segment leaves to estimate damage—a task that requires a spatial understanding of the diversity of leaf shapes and sizes—it is more straightforward and more precise to estimate leaf damage on leaves with lower levels of damage, especially if the damage is located on the leaf margins. Leaf margin damage was also misestimated by artificial intelligence methods like the HerbiEstim (Wang et al., 2024) because this tool was unable to reconstruct the leaf image and calculate the amount of tissue loss in leaves with higher herbivory or with higher damage on the leaf margins. Contrary to our expectations, leaf size had no effect on the delta herbivory between visual and image analysis methods. Therefore, regardless of leaf size, researchers are likely to overestimate herbivory levels using visual methods. However, considering that the visual estimation method is quicker and becomes more accurate with proper training, we suggest that it can be used for leaves with low levels of herbivory to expedite measurements. For leaves with higher levels of damage, digital methods should be employed to ensure accuracy. Because our results also indicate that both ImageJ and EBImage consistently measure herbivory levels with similar accuracy for leaves with natural herbivory, and also no differences in herbivory accuracy were found for leaves with artificial herbivory using the three digital methods, any of these methods can be used with similar results and efficiency.

As expected, the time required to perform the measurements varied among the methods. Visual estimates took ~15 times less time than the other digital methods, and the visual method can be advantageous when robust accuracy of herbivory levels is not necessary, such as in relative damage comparisons made by the same researcher across different plant species simultaneously. Conversely, in some situations, a substantially larger sample size, though with less accuracy per leaf, could be important to the goals of a study and could yield more meaningful data than more accurate estimates for a smaller number of leaves. To do so, one can estimate herbivory on all leaves within a delimited canopy volume (Ribeiro & Basset, 2007), or from a large number of randomly sampled small plant individuals, producing the average leaf herbivory for each volume/sampling unit, and having many repetitions. Such distribution of means may be appropriate to deal with community‐scale hypotheses, but also could be used to counter‐calibrate more accurate, but sampled/time‐constrained methods. By knowing the difference in accuracy of visual and digital methods, one may use a small pilot with both to produce a linear regression correction coefficient, facilitating comparison with the literature based on other methods. Also, the use of devices that might replace human estimation in the field, with adequate image‐capturing mechanisms, could be a future interesting contribution for a balance between sample size, precision, and exactitude (Silva et al., 2021).

Interestingly, the time spent to measure leaves with the EBImage package in the R software was longer, averaging 13 more minutes for each set of 20 leaves compared to ImageJ. We had predicted that measurements in EBImage would be faster than in ImageJ due to its automation capabilities, allowing researchers to measure leaves in several images or even entire folders of digital images of several species in a few minutes, provided they are on the same scale (in number of pixels in 1 cm^2^). EBImage also automatically saves the measurements in a spreadsheet, eliminating the possibility of transcription errors. The observed time difference between these two methods likely occurred during the image preparation step rather than the measurement time. The measurements in EBImage in R were made by 18 researchers, many of whom were unfamiliar with this method. Therefore, we developed a detailed protocol aiming at minimizing methodological biases and facilitating image processing to save time (see Appendix S1: Section S1).

We predict that broader use of the EBImage package with various leaf sets will facilitate future comparisons with commonly used software today (e.g., ImageJ), and with mobile applications such as BioLeaf (Machado et al., 2016) and LeafByte (Getman‐Pickering et al., 2020). Additionally, comparisons can be made with deep‐learning algorithms like HerbiEstim (Wang et al., 2024). Although deep learning and automatic estimation of foliar herbivory have become recently available (see Vieira et al., 2022, 2025; Wang et al., 2024) and can turn into a very useful tool in the near future, precise data analyses using AI‐based tools are yet to be demonstrated. Some of these applications have only been tested on cultivars where the average damage and leaf traits are well known (see Vieira et al., 2025). Furthermore, some AI tools are calibrated using species‐specific models developed for a limited number of plant species. Our study was limited to Neotropical plants, which may differ in leaf traits and herbivory patterns from temperate or other tropical regions. For instance, thicker leaves or specific herbivore guilds in other biomes could influence the performance of visual or digital methods. Future cross‐biome comparisons would help evaluate the generalizability of our recommendations.

Considering the advantages and disadvantages discussed above, we recommend using software based on digital images of leaves (e.g., ImageJ, EBImage) for herbivory estimation when the goals of the study are highly accurate herbivory estimates for comparison of leaves or plants that have similar levels of herbivory. The visual method, in contrast, is most suited to answering questions that require large sample sizes and when the focus is on describing broad patterns and major differences in damage levels, and it is important that researchers are trained in visual estimation using tools like the Zax Herbivory Trainer.

### Comparisons between image data and leaf traits

As predicted, we observed differences in the time required to estimate leaf area loss between leaves of varying sizes and across different herbivory levels. Because we did not detect differences in herbivory measurements between colored and black‐and‐white images, nor differences in the time required for herbivory measurements for either image color, we recommend using color images. Color images reduce the number of steps needed for image processing and allow for a more detailed evaluation of leaf shadows, blemishes, and missing areas during image processing, increasing accuracy in herbivory detection. We also recommend digitizing oven‐dried, pressed leaves for image‐based and software methods usage. Researchers must consider, however, the time required for such processing, often done in the field (pressing) and in the laboratory (oven drying), as well as the volume of collected material and storage space.

Regarding image source, the lack of differences in herbivory measurements between image acquisition techniques suggests the use of digitized images with a flatbed scanner instead of a digital camera. As expected with photographs, the scanner reduces measurement errors mainly related to scales and reduces image distortions and shadow production. Given the lack of differences in herbivory accuracy between the number of leaves per image, we recommend using several leaves per image to reduce the time required to estimate herbivory levels. We also suggest making measurements not only on multiple leaves per image but also on multiple folders containing the entire set of leaves to be analyzed, especially if using EBImage, thus reducing the total measurement time. Despite no differences in herbivory measurements between leaf faces, we recommend using the abaxial face due to the central vein potentially preventing the leaf from being completely flat during image capture, thus creating shadows that can easily mask leaf herbivory marks or be mistaken for the total leaf area.

## CONCLUSION

Our study comprehensively analyzes both traditional and contemporary methods for quantifying herbivory across a diverse range of tropical species. We found that both visual estimates and deep machine learning introduced biases, overestimating and underestimating herbivory levels, respectively. ImageJ and EBImage offered a reliable and somewhat indistinguishable approach to quantifying herbivory based on digital images of leaves, especially for damage dispersed over the center of the leaf blade. Despite the simplicity, speed, and widespread use of visual estimation, our findings suggest that this method should be used cautiously and after researchers undergo training to recognize and quantify herbivory more precisely. In light of our findings, particularly the overestimation revealed by the artificial herbivory experiment, we recommend that researchers prioritize digital image analysis for the majority of ecological studies where accurate quantification of herbivory is the goal.

Methodological biases are present in both visual and digital methods. However, these biases can be minimized through standardized and transparent protocols. By adopting such protocols, researchers can enhance the accuracy of herbivory quantification—improving our understanding of plant–herbivore interactions and their implications for plant population dynamics, community organization, and ecosystem processes. Our study underscores the importance of carefully selecting methods for herbivory quantification depending on the trade‐off between time and accuracy, and highlights the need for training and for the use of standardized protocols in herbivory studies. We hope this work will help investigators select methods that will work best for the aims of their research and use those methods most effectively, thereby improving the accuracy and precision of the field's understanding of leaf herbivory across ecological, geographical, and phylogenetic gradients.

## CONFLICT OF INTEREST STATEMENT

The authors declare no conflicts of interest.

## Supporting information


Appendix S1.

## Data Availability

Data and code (Cornelissen, 2024) are available in Figshare at https://doi.org/10.6084/m9.figshare.27123372.v1.

## References

[ecy70308-bib-0001] Abràmoff, M. D. , P. J. Magalhães , and S. J. Ram . 2004. “Image Processing with imageJ.” Biophotonics International 11: 36–41.

[ecy70308-bib-0002] Alliende, M. C. 1989. “Demographic Studies of a Dioecious Tree. II. The Distribution of Leaf Predation within and between Trees.” Journal of Ecology 77: 1048.

[ecy70308-bib-0003] Bakker, E. S. , K. A. Wood , J. F. Pages , G. F. Veen , M. J. A. Christianen , L. Santamaria , B. A. Nolet , and S. Hilt . 2016. “Herbivory on Freshwater and Marine Macrophytes: A Review and Perspective.” Aquatic Botany 135: 18–36.

[ecy70308-bib-0004] Bigger, D. S. , and M. A. Marvier . 1998. “How Different Would a World without Herbivory be? A Search for Generality in Ecology.” Integrative Biology: Issues, News, and Reviews 1: 60–67.

[ecy70308-bib-0005] Cébrian, J. , and C. M. Duarte . 2016. “Patterns in Leaf Herbivory on Seagrasses.” Aquatic Botany 60: 67–82.

[ecy70308-bib-0006] Cornelissen, T. 2024. “Files Associated to the Handbook of Herbivory Quantification.” figshare. Dataset. 10.6084/m9.figshare.27123372.v1.

[ecy70308-bib-0007] Díaz, M. , A. P. Møller , and F. J. Pulido . 2003. “Fruit Abortion, Developmental Selection and Developmental Stability in Quercus Ilex.” Oecologia 135: 378–385. 12721827 10.1007/s00442-003-1202-y

[ecy70308-bib-0008] Dirzo, R. , and C. A. Domínguez . 1995. “Plant–Herbivore Interactions in Mesoamerican Tropical Dry Forests.” In Seasonally Dry Tropical Forests, edited by S. H. Bullock , H. A. Mooney , and E. Medina , 304–325. Cambridge: Cambridge University Press.

[ecy70308-bib-0009] Emer, C. , N. Villar , N. Melo , V. B. Ziparro , S. Nazareth , and M. Galetti . 2024. “The Interplay between Defaunation and Phylogenetic Diversity Affects Leaf Damage by Natural Enemies in Tropical Plants.” Journal of Ecology 112: 971–984.

[ecy70308-bib-0010] Frost, C. J. 2022. “A Visual Technique Used by Citizen Scientists Shows Higher Herbivory in Understory Vs. Canopy Leaves of a Tropical Forest.” Ecology 103: 1–8. 10.1002/ecy.3539 34582569

[ecy70308-bib-0011] Futuyma, D. J. , and A. A. Agrawal . 2009. “Macroevolution and the Biological Diversity of Plants and Herbivores.” Proceedings of the National Academy of Sciences of the United States of America 106: 18054–18061. 19815508 10.1073/pnas.0904106106 PMC2775342

[ecy70308-bib-0012] Getman‐Pickering, Z. L. , A. Campbell , N. Aflitto , A. Grele , J. K. Davis , and T. A. Ugine . 2020. “LeafByte: A Mobile Application that Measures Leaf Area and Herbivory Quickly and Accurately.” Methods in Ecology and Evolution 11: 215–221.

[ecy70308-bib-0013] González‐Browne, C. , M. M. Murúa , L. Navarro , and R. Medel . 2016. “Does Plant Origin Influence the Fitness Impact of Flower Damage? A Meta‐Analysis.” PLoS One 11: e0146437. 26785039 10.1371/journal.pone.0146437 PMC4718695

[ecy70308-bib-0014] Janzen, D. H. 1981. “Patterns of Herbivory in a Tropical Deciduous Forest.” Biotropica 13: 271–282.

[ecy70308-bib-0015] Johnson, M. T. J. , J. A. Bertrand , and M. M. Turcotte . 2016. “Precision and Accuracy in Quantifying Herbivory.” Ecological Entomology 41: 112–121.

[ecy70308-bib-0016] Katabuchi, M. 2015. “LeafArea: An R Package for Rapid Digital Image Analysis of Leaf Area.” Ecological Research 30: 1073–1077.

[ecy70308-bib-0017] Kozlov, M. V. , V. Lanta , V. Zverev , and E. L. Zvereva . 2015a. “Background Losses of Woody Plant Foliage to Insects Show Variable Relationships with Plant Functional Traits across the Globe.” Journal of Ecology 103: 1519–1528.

[ecy70308-bib-0018] Kozlov, M. V. , V. Lanta , V. Zverev , and E. L. Zvereva . 2015b. “Global Patterns in Background Losses of Woody Plant Foliage to Insects.” Global Ecology and Biogeography 24: 1126–1135.

[ecy70308-bib-0019] Kozlov, M. V. , V. Zverev , and E. L. Zvereva . 2014. “Confirmation Bias Leads to Overestimation of Losses of Woody Plant Foliage to Insect Herbivores in Tropical Regions.” PeerJ 2: e709. 25551025 10.7717/peerj.709 PMC4277485

[ecy70308-bib-0020] Kozlov, M. V. , and E. L. Zvereva . 2017. “Background Insect Herbivory: Impacts, Patterns and Methodology.” In Progress in Botany, edited by F. M. Cánovas , U. Lüttge , and R. Matilde , vol. 78, 313–355. Cham: Springer International Publishing.

[ecy70308-bib-0021] Lincoln, D. E. , and H. A. Mooney . 1984. “Herbivory on Diplacus Aurantiacus Shrubs in Sun and Shade.” Oecologia 64: 173–176. 28312335 10.1007/BF00376867

[ecy70308-bib-0022] Liu, M. , P. Jiang , J. M. Chase , and X. Liu . 2024. “Global Insect Herbivory and Its Response to Climate Change.” Current Biology 34: 2558–2569.e3. 38776900 10.1016/j.cub.2024.04.062

[ecy70308-bib-0023] Louda, S. M. 1984. “Herbivore Effect on Stature, Fruiting, and Leaf Dynamics of a Native Crucifer.” Ecology 65: 1379–1386.

[ecy70308-bib-0024] Machado, B. B. , J. P. M. Orue , M. S. Arruda , C. V. Santos , D. S. Sarath , W. N. Goncalves , G. G. Silva , H. Pistori , A. R. Roel , and J. F. Rodrigues‐Jr . 2016. “BioLeaf: A Professional Mobile Application to Measure Foliar Damage Caused by Insect Herbivory.” Computers and Electronics in Agriculture 129: 44–55.

[ecy70308-bib-0025] Marquis, R. J. 1984. “Leaf Herbivores Decrease Fitness of a Tropical Plant.” Science 226: 537–539. 17821511 10.1126/science.226.4674.537

[ecy70308-bib-0026] Mendes, G. M. , F. A. O. Silveira , C. Oliveira , W. Dáttilo , R. Guevara , B. Ruiz‐Guerra , M. G. Boaventura , et al. 2021. “How Much Leaf Area Do Insects Eat? A Data Set of Insect Herbivory Sampled Globally with a Standardized Protocol.” Ecology 102: 3301. 10.1002/ecy.3301 33565639

[ecy70308-bib-0046] Oleś, A. , G. Pau , F. Fuchs , O. Sklyar , M. Boutros , and W. Huber . 2024. “EBImage: Image Processing and Analysis Toolbox for R.” R Package Version 4.46.0.

[ecy70308-bib-0027] Olivoto, T. 2022. “Lights, Camera, Pliman! An R Package for Plant Image Analysis.” Methods in Ecology and Evolution 13: 789–798.

[ecy70308-bib-0028] Pau, G. , F. Fuchs , O. Sklyar , M. Boutros , and W. Huber . 2010. “EBImage‐an R Package for Image Processing with Applications to Cellular Phenotypes.” Bioinformatics 26: 979–981. 20338898 10.1093/bioinformatics/btq046 PMC2844988

[ecy70308-bib-0029] Pinheiro, J. , D. Bates , and R Core Team . 2025. “nlme: Linear and Nonlinear Mixed Effects Models.” R Package Version 3.1: 168.

[ecy70308-bib-0030] Price, P. W. 2002. “Resource‐Driven Terrestrial Interaction Webs.” Ecological Research 17: 241–247.

[ecy70308-bib-0031] Qian, H. , and Y. Jin . 2016. “An Updated Megaphylogeny of Plants, a Tool for Generating Plant Phylogenies and an Analysis of Phylogenetic Community Structure.” Journal of Plant Ecology 9: 233–239.

[ecy70308-bib-0032] R Core Team . 2023. R: A Language and Environment for Statistical Computing. Vienna: R Foundation for Statistical Computing.

[ecy70308-bib-0033] Revell, L. J. 2012. “phytools: An R Package for Phylogenetic Comparative Biology (and Other Things).” Methods in Ecology and Evolution 3: 217–223.

[ecy70308-bib-0034] Ribeiro, S. P. , and Y. Basset . 2007. “Gall‐Forming and Free‐Feeding Herbivory along Vertical Gradients in a Lowland Tropical Rainforest: The Importance of Leaf Sclerophylly.” Ecography 30: 663–672.

[ecy70308-bib-0035] Schneider, C. A. , W. S. Rasband , and K. W. Eliceiri . 2012. “NIH Image to ImageJ: 25 Years of Image Analysis.” Nature Methods 9: 671–675. 22930834 10.1038/nmeth.2089 PMC5554542

[ecy70308-bib-0036] Silva, M. C. , J. C. F. da Silva , S. Delabrida , A. G. C. Bianchi , S. P. Ribeiro , J. S. Silva , and R. A. R. Oliveira . 2021. “Wearable Edge AI Applications for Ecological Environments.” Sensors (Basel) 21: 5082. 34372319 10.3390/s21155082 PMC8347733

[ecy70308-bib-0037] The Herbivory Variability Network . 2023. “Plant Size, Latitude, and Phylogeny Explain within‐Population Variability in Herbivory.” Science 382: 679–683. 37943897 10.1126/science.adh8830

[ecy70308-bib-0038] Turcotte, M. M. , T. J. Davies , C. J. M. Thomsen , and M. T. J. Johnson . 2014. “Macroecological and Macroevolutionary Patterns of Leaf Herbivory across Vascular Plants.” Proceedings of the Royal Society B: Biological Sciences 281: 20140555. 10.1098/rspb.2014.0555 PMC4071545 24870043

[ecy70308-bib-0039] Turcotte, M. M. , C. J. M. Thomsen , G. T. Broadhead , P. V. A. Fine , R. M. Godfrey , G. P. A. Lamarre , S. T. Meyer , L. A. Richards , and M. T. J. Johnson . 2014. “Percentage Leaf Herbivory across Vascular Plant Species.” Ecology 95: 788.

[ecy70308-bib-0040] Ullah, M. I. , M. Arshad , S. Ali , A. Abdullah , S. Khalid , H. M. Aatif , S. M. A. Zahid , M. Afzal , and J. Molina‐Ochoa . 2020. “Using Smartphone Application to Estimate the Defoliation Caused by Insect Herbivory in Various Crops.” Pakistan Journal of Zoology 52: 1129–1135.

[ecy70308-bib-0041] Vieira, G. S. , A. U. Fonseca , N. M. de Sousa , J. C. Ferreira , J. P. Felix , C. D. Cabacinha , N. M. de Sousa , and F. Soares . 2025. “An Automatic Method for Estimating Insect Defoliation with Visual Highlights of Consumed Leaf Tissue Regions.” Information Processing in Agriculture 12: 40–53.

[ecy70308-bib-0042] Vieira, G. S. , A. U. Fonseca , B. M. Rocha , N. M. Sousa , J. C. Ferreira , J. P. Felix , J. C. Lima , and F. Soares . 2022. “Insect Predation Estimate Using Binary Leaf Models and Image‐Matching Shapes.” Agronomy 12: 1–15.

[ecy70308-bib-0043] Wang, Z. , Y. Jiang , A. B. Diallo , and S. W. Kembel . 2024. “Deep Learning‐ and Image Processing‐Based Methods for Automatic Estimation of Leaf Herbivore Damage.” Methods in Ecology and Evolution 15: 732–743.

[ecy70308-bib-0044] Wetzel, W. C. , B. D. Inouye , P. G. Hahn , S. R. Whitehead , and N. Underwood . 2023. “Variability in Plant–Herbivore Interactions.” Annual Review of Ecology, Evolution, and Systematics 54: 451–474.

[ecy70308-bib-0045] Xirocostas, Z. A. , S. A. Debono , E. Slavich , and A. T. Moles . 2022. “The ZAX Herbivory Trainer—Free Software for Training Researchers to Visually Estimate Leaf Damage.” Methods in Ecology and Evolution 13: 596–602.

