# Supplementary material for: Quantifying leaf herbivory: A guide to methodological trade‐offs and best practices
Source: Ecology. 2026 Feb 3;107(2):e70308. doi: 10.1002/ecy.70308 (PMC12867601; doi:10.1002/ecy.70308)
Supplement: Supplementary file 1 — Appendix S1. [file ECY-107-e70308-s001.pdf]

Ecology

Appendix S1

Quantifying leaf herbivory: A guide to methodological trade-offs and best practices

Tatiana Cornelissen, Gisele M. Mendes, Fernando A. O. Silveira, Wesley Dáttilo, Roger Guevara, Ramiro Aguilar, Maria Gabriela Boaventura, Ricardo Campos, Ek del Val, Guilherme Ramos Demetrio, Marcilio Fagundes, Rafael de Paiva Farias, Geraldo W. Fernandes, Tiago Fernandes, Inácio Gomes, Thiago Kloss, Juliana Kuchenbecker, Leandro Maracahipes, Frederico Neves, Lucas Paolucci, Cássio Cardoso Pereira, Elenir Queiroz, Letícia Ramos, Sérvio P. Ribeiro, Gustavo Q. Romero, Carolina Oliveira, Jhonathan O. Silva, Tathiana Sobrinho, Ricardo Solar, Heraldo Vasconcelos, Gabriela Zorzal, William C. Wetzel

.

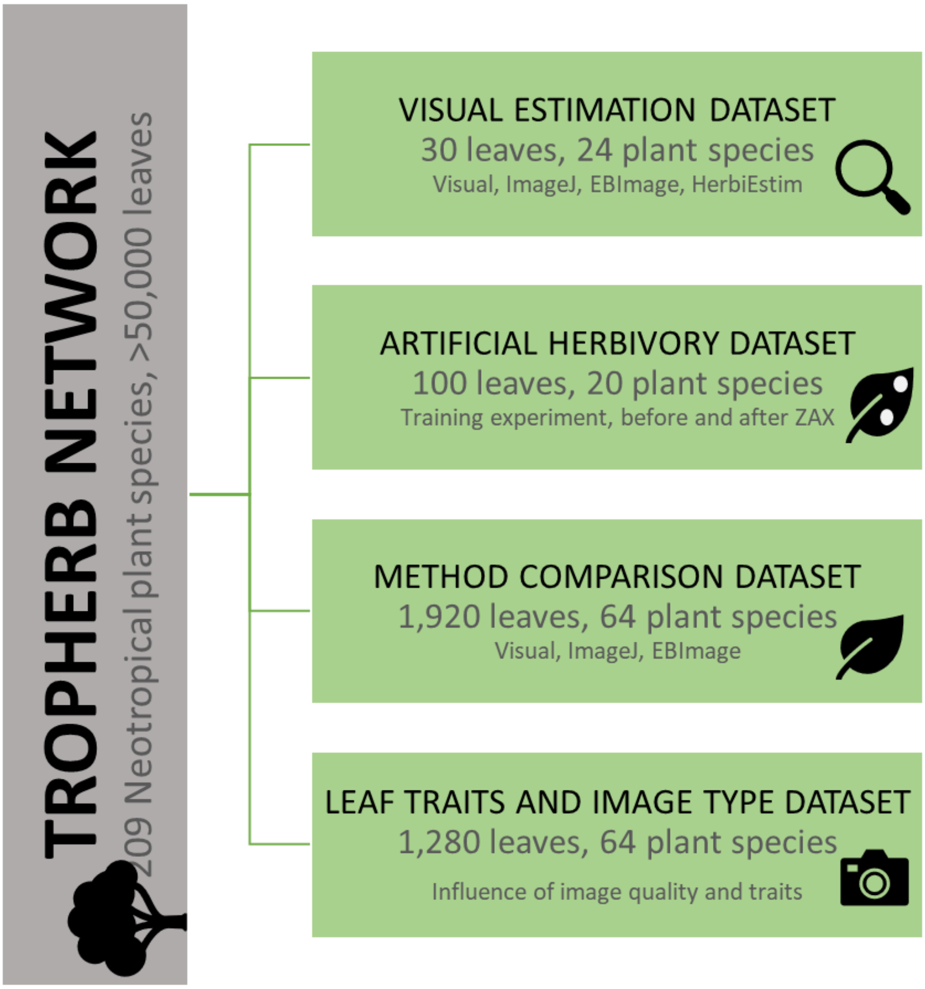

Figure S1. Schematic representation of the datasets used for each one of the analysis and comparisons of methods to estimate leaf herbivory. Data were derived from a large dataset from the TropHerb network and number of plant species and leaves evaluated for each subgroup are listed within the rectangles. Figure created by Tatiana Cornelissen using icons from Microsoft PowerPoint.

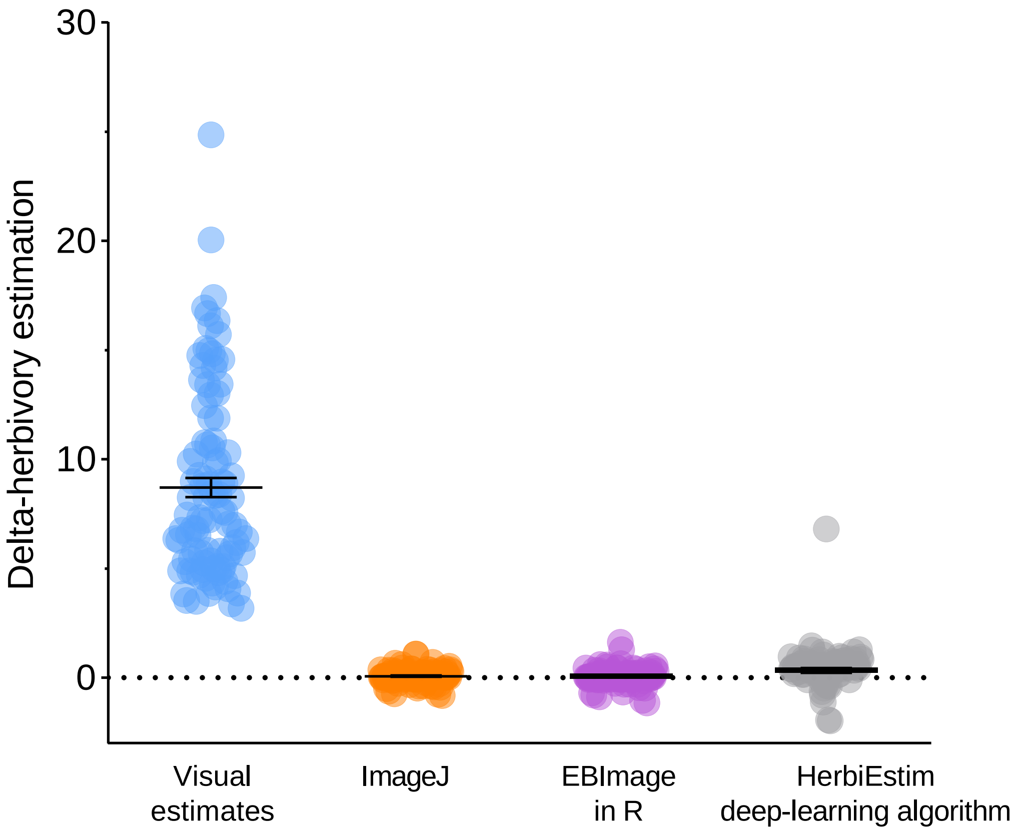

Figure S2. Delta-herbivory estimation (difference between estimation of herbivory and actual herbivory level in plants created after punching holes of known area in intact leaves) among the four different methods. Please see text for explanation of each method. (Visual estimates: 8.709 ± 0.42; ImageJ: 0.068 ± 0.031; EBImage: 0.064 ± 0.037; HerbiEstim: 0.335 ± 0.112; Mean ± standard error).

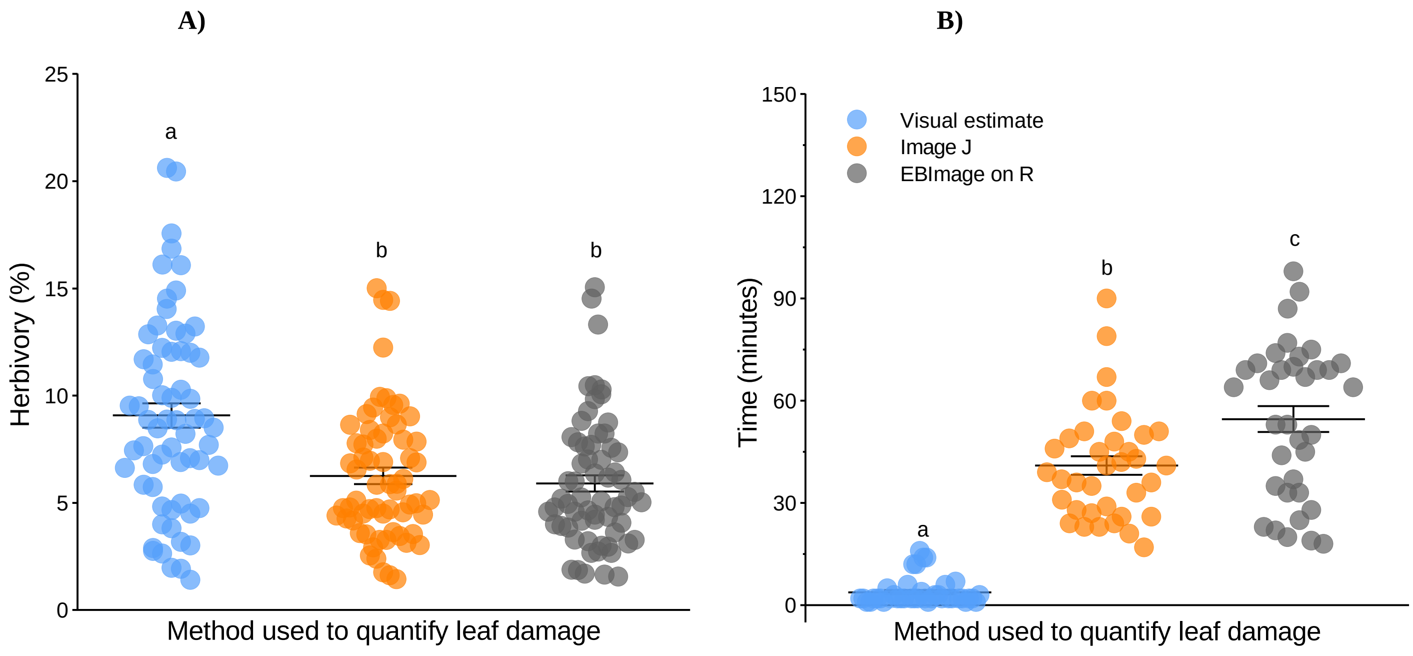

Figure S3. A) Herbivory level (mean ± SE) of 64 plant species as a function of method used to quantify leaf damage and B) Time spent, in minutes, to estimate herbivory level as a function of method used. Different letters indicate significant differences between methods, according to contrast analysis after GLMM. Each point represents a plant.

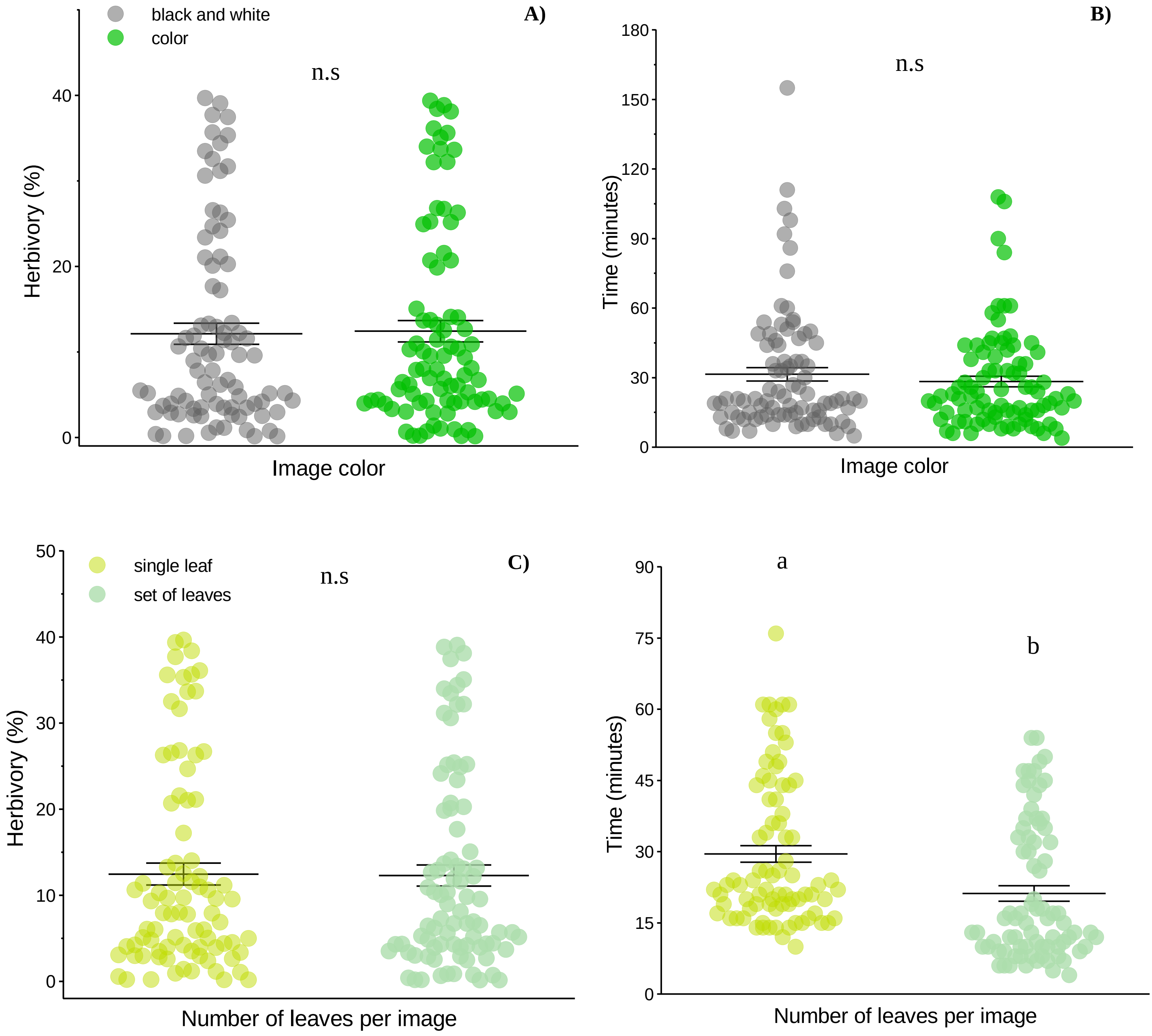

Figure S4. A) Herbivory estimates (mean ± SE) as a function of image color (black and white n=85 and color image n=85), B) Time spent, in minutes, to estimate herbivory according to leaf color image, C) Herbivory estimates (mean ± SE) as a function of the number of leaves in the image file (n=85 on each category), D) Time spent, in minutes, to estimate herbivory according to the number of leaf images in each image file. Each point represents an image. Significant differences at α=0.05 are indicated by different letters. n.s.= non-significant.

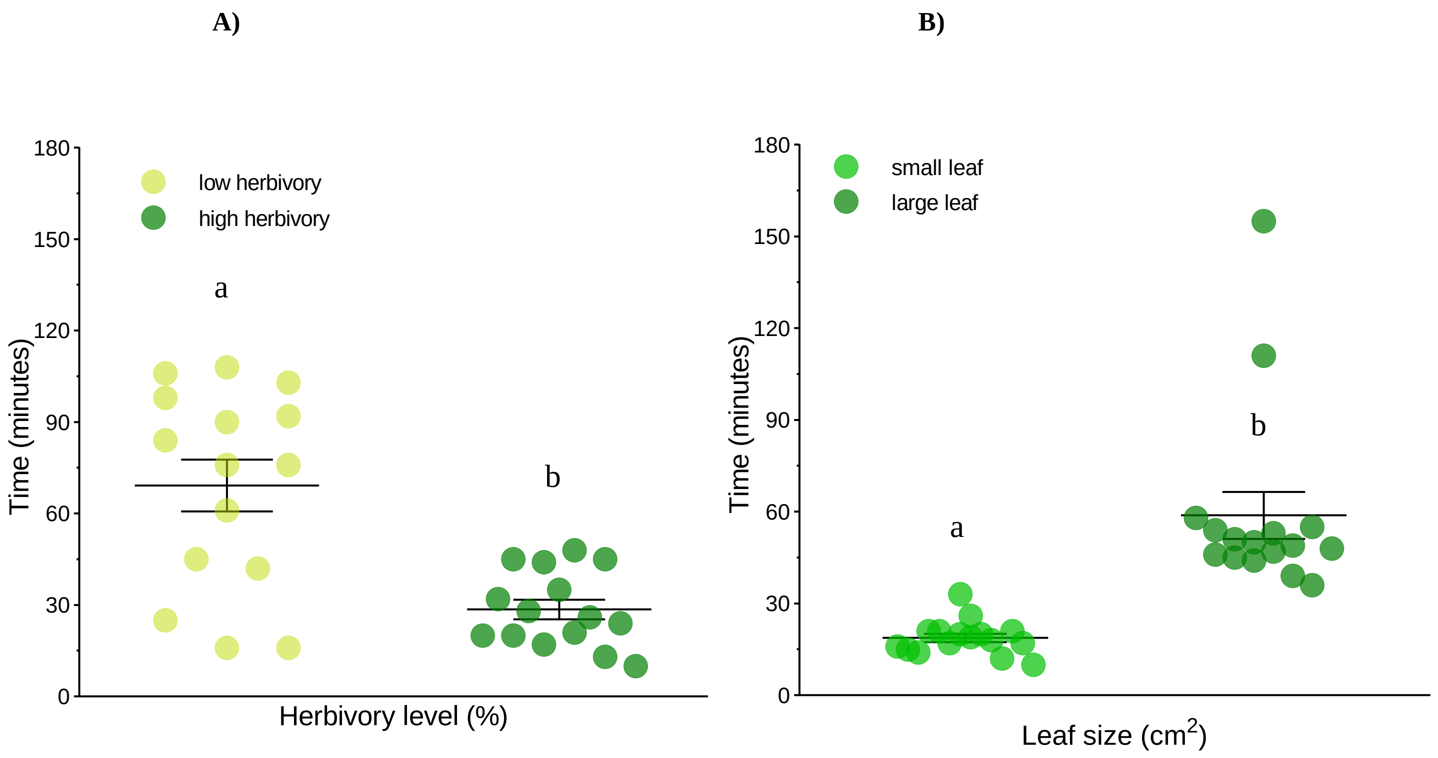

Figure S5. A) Time spent (mean ± SE), in minutes, to estimate leaf area lost according to foliar leaf herbivory level (low herbivory level: less than 6% of leaf area removed (n= 15) and high herbivory level: more than 25% of leaf area removed (n= 15). B) Time spent (mean ± SE), in minutes, to estimate leaf area lost according to leaf size (small leaf: up to 6 cm^2 (n= 34) and large leaf: larger than 60 cm^2 (n= 16). Each point represents a leaf. Significant differences at α=0.05 are indicated by different letters.

Section S1

The proliferation of different methods to estimate herbivory on land plants prevents cross-study comparisons and impairs biogeographical and macroecological approaches to understand multiple ecological and evolutionary dimensions of plant-insect interactions. This handbook aims to provide general guidelines to quantify leaf herbivory on plants under field conditions to allow comparisons among studies, improve reproducibility and accuracy of measurements, and reduce bias of herbivory levels in plants. This protocol is divided into two major steps. Step A provides information on data collection and Step B provides detailed information on image processing and storing. To obtain standardized measurements of additional leaf traits, please refer to Pérez-Harguindeguy *et al.* (2013).

Step A. DATA COLLECTION

1. **Site selection**
   Samples should be preferably taken in natural areas. Please make sure to report site coordinates (latitude and longitude) and elevation range. Select sites that are representative of the biome or habitat and report the level of human impact, if necessary.

2. **Species selection**
   Species selection criteria fundamentally depend on the study goal. A general protocol suggests data to be collected on the five most abundant plant species to allow characterization of leaf area removed by herbivores at a given site (Mendes et al. 2021). Randomly select and tag five individuals of each species.

3. **Sampling leaves**
   For each individual, randomly select one branch for sampling. Under the impossibility of random selection, chose one representative branch from a distance of two meters from the individual. From each branch, sampled a maximum of 50 leaves. Each individual leaf should be numbered, pressed and oven-dried at 45 C^{o} for 48 to 72 hours. Number the 50 leaves from each plant replicate with a four-digit code that indicate the species, the individual plant and the leaf (example: *Bauhinia brevipes* should be Bb 1.1 to 1.50, BB 2.1 to 2.50…Bb 5.1 to 5.50). If you cannot write the code with a permanent pen on the leaf blade, attach a piece of tape to the petiole and write the code in the tape with a permanent pen.
   The use of this protocol yields a sample size of 1,250 leaves per locality (five species x five replicates x one branch per replicate x 50 leaves/branch).

4. **Leaf scanning and image storage**

   Digitize the leaves using a high-quality flatbed scanner, with color images and a resolution of at least 300 dpi, in A4 or Letter size paper. Include a plastic or metal ruler (in cm or mm) to serve as a scale reference in each scanned image. Preferably, opt for the abaxial side of the leaves and fit as many leaves as possible in each image. For each species, save one color image containing all 50 leaves (e.g., Bauhinia_brevipes_all). For plants with large leaves that cannot fit into a single image, save the images as follows: (e.g., Bauhinia_brevipes1; Bauhinia_brevipes2; Bauhinia_brevipes3). Organize the images into separate folders by species; name them in a simple and clear manner. The folder should contain only the leaves to be measured (e.g., original_photos_plantnameXXX).

5. **Unit of measurement of herbivory**
   Leaf herbivory is expressed by the ratio between sum of leaf area losses over the leaf lamina by total leaf area, multiplied by 100 to convert into percentage. This protocol allows for assessments of leaf herbivory at the leaf and plant scales.

Step B. IMAGE PROCESSING

Required software: GIMP 2.1®

Required storage: 4 GB of free space on the hard drive.

Files for analysis: A folder containing the photos to be processed

For the purposes of this handbook, a set of leaves is composed of 50 leaves sampled from the same individual plant.

For each original individual image, process and save one color image containing all 50 leaves, one black and white image containing all 50 leaves, 50 images each containing one of the leaves from the original color image, and 50 images each containing one of the leaves from the original black and white image.

#Step 1

# Colored images (set of 50 leaves, one or more plates per species)

1. **Importing the image**
   Open the GIMP 2.1 program → right-click → File → open → navigate to the project folder, open the folder "original_photos_plantname.XXX," and double-click to open the first photo.

2. **Deleting image background**
Click on contiguous selection tool (Figure S6) → click on any white area of the image and press delete. This should delete all the white background of the image. Click on the rectangular selection tool (shortcut "R") and click on any area of the photo to reselect the entire image.

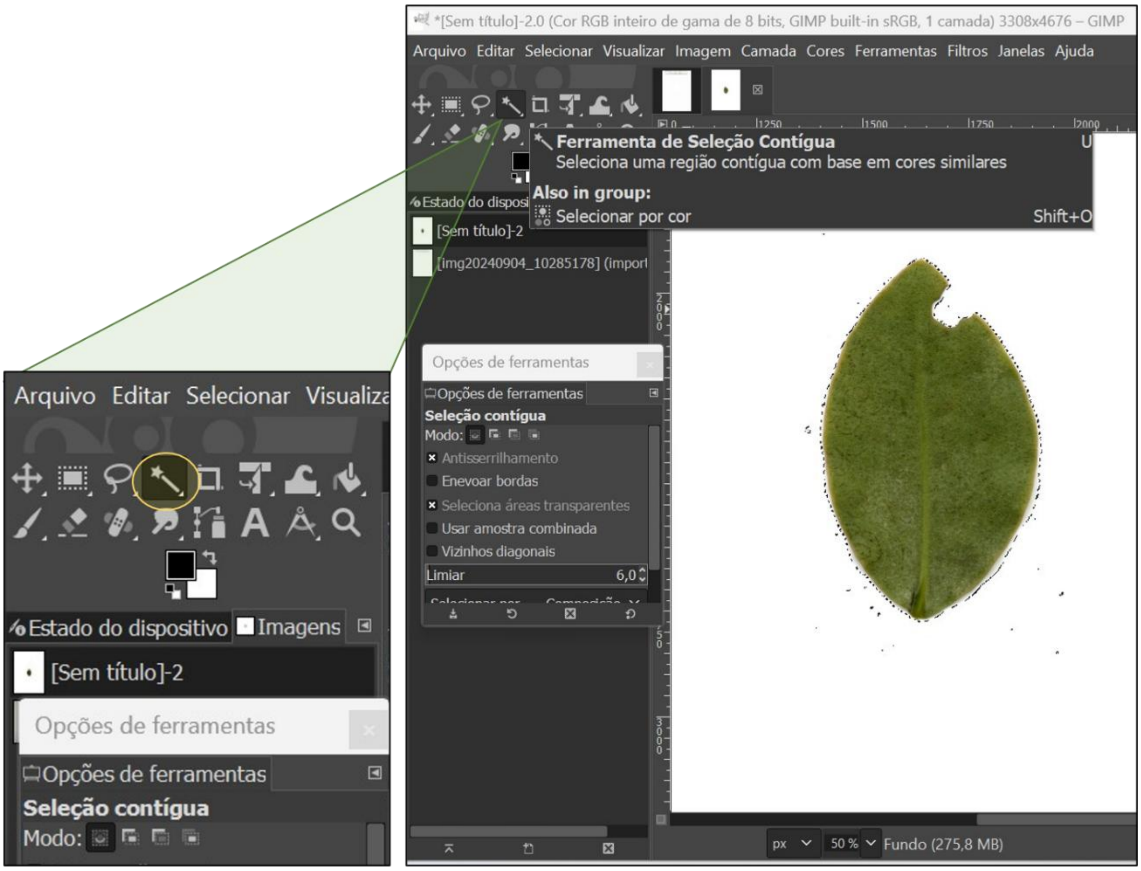

Figure S6. Highlighted, contiguous selection tool in ImageJ (Photo credit: Gisele M. Mendes).

3. **Deleting other information (name, scale, legend, etc.)**
Use the rectangular selection tool (shortcut "R") to select the area where the scales and numbering are located and delete.

Note: If shadows (dark areas) are present on leaf edges, also select and delete these shadows as they may interfere with the subsequent image analysis.

4. **Drawing the outline of each individual leaf**
Delimit the entire set of 50 leaves for each species. Click on the pencil tool in the toolbox on the left side of the screen (shortcut key "N") and select the color black → in the settings panel that appears below the tools. Pencil thickness

"size" should be adjusted to value 2 and then draw the leaf boundaries (see Figure S7).

Note: Exclude the petiole from all leaves during measurements.

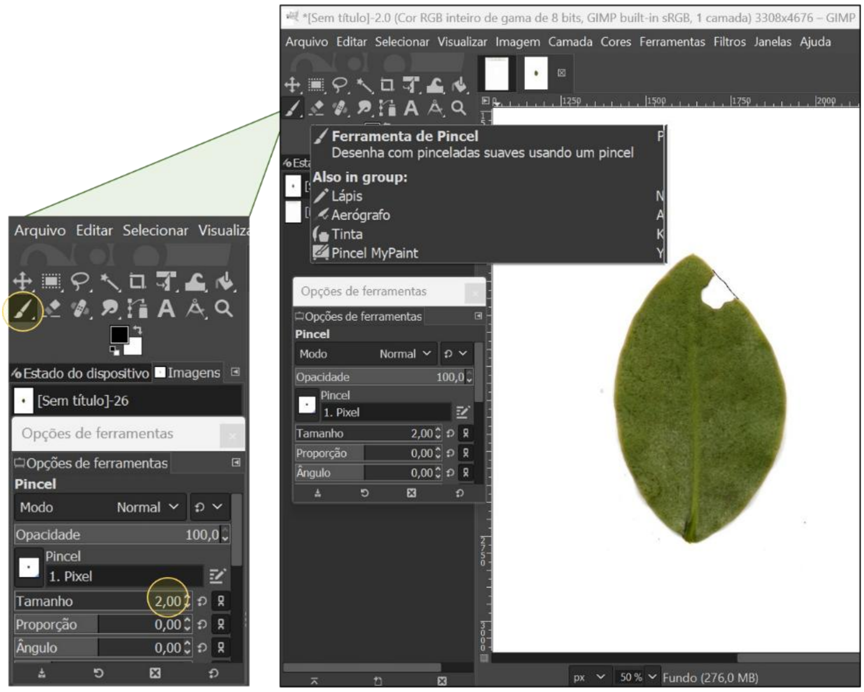

Figure S7. Example of a leaf which margin was redrawn before herbivory measurement can be taken (Photo credit: Gisele M. Mendes).
.

#Step 2

# Exporting the image

1. **Exporting images**
   Export the **colored image**. Click on the right button → "File" → "Export as..." (shortcut CONTROL + SHIFT + E) → modify the photo name following the pattern:

plantname.identificationoftheplate(letter).color.png

E.g.: Bforticata.A.color.png

Create a new folder named plants.color by clicking "Create Folder". If it does not already exist then choose the folder and click "Export". In the settings box that will

appear, mark only "Save resolution" and "Save color profile" and click export. Check if the file was saved correctly.

Note 1: Always save files as .png
Note 2: The file name should not contain spaces, use only "_" if necessary.
Note 3: Save all plate images (complete and colored) in this folder.

Example: Bforticata.A.color.png

#Step 3

# Black and white image (set of 20 leaves, one or more plates per species)

1. **Converting all leaves to black and white.**
   Using the processed image by right-clicking on colors, then threshold, and finally click on spreadview. Drag the line to check the herbivory areas and leaf boundaries and if necessary, adjust the threshold level.

2. **Exporting images**

   Export the black/white image. Right-click on "File" then "Export as..." (shortcut CONTROL + SHIFT + E) → change image name following the pattern:

   plantname.identificationoftheplate(letter).BW.png

   EX: Bforticata.A.BW.png

   Create a new folder named **plants.BW** by clicking on "Create Folder", if it does not already exist → Choose the folder, click "Export" and leave only "Save resolution" and "Save color profile" checked in the settings box. Finally, click export and check whether the file was saved correctly.

   Note: Always save files as .png
   Note 2: The file name should not contain spaces, use only "_" if necessary.
   Note 3: Save all images (complete and black/white) in this folder.

   Example: Bforticata.A.BW.png

#Step 4
# One leaf per image (color, 20 images per species)

1. **Return to the color image: Control+Z**

2. **Leaf processing order**
   Sequence the leaves from 1 to 20 (Figure S8), always from left to right and from top to bottom.

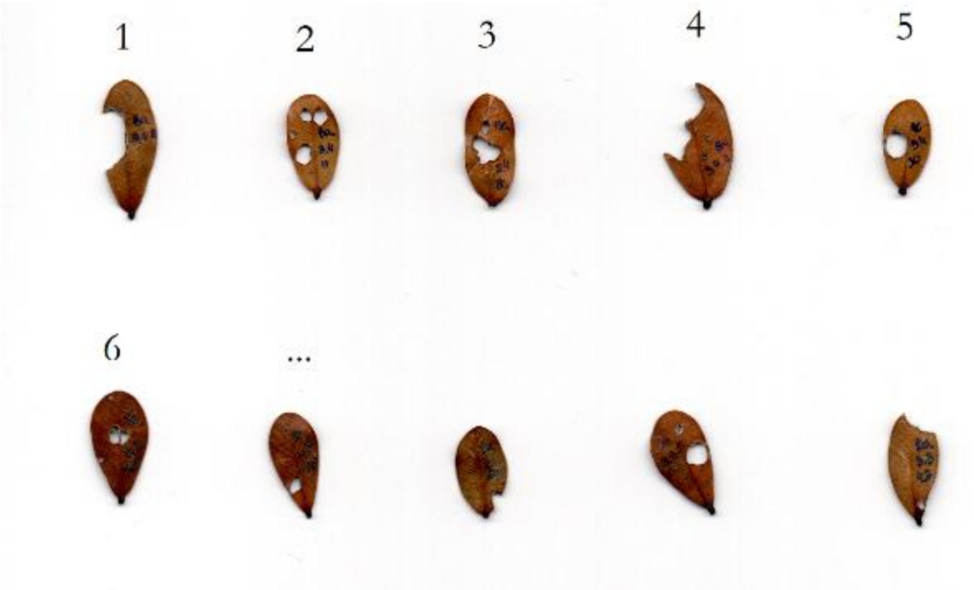

Figure S8. Example of sequence of measurements for herbivory estimation. Leaves should be numbered and measured from left to right and top to bottom (Photo credit: Gisele M. Mendes).

3. **Save each of the leaves as separate images**
   Use the rectangular selection tool (shortcut key: r) to select leaf 1, then Ctrl + X to cut and Ctrl + N to generate a new file. At this stage, after making sure the size/pixels of the new file are consistent with the original file, enter Ctrl + V to paste.

4. **Exporting images**
   Export the color image. Right-click on "File", "Export as..." (shortcut CONTROL + SHIFT + E) → modify the photo name following the pattern: plantname.leafnumber.color.png

E.g.: Bforticata.1.color.png

Create a new folder named **plantname.color** by clicking "Create Folder", if it does not already exist choose the folder, click "Export" and leave only "Save resolution" and "Save color profile" checked in the settings box. Then click export and heck whether the file was saved correctly.

Note: Always save files as .png
Note 2: The file name should not contain spaces, use only "_" if necessary.
Note 3: Save all images (complete and black/white) in this folder.

3. **Repeat the above process for all individual leaves (total = 50 leaves per individual plant, 5 plants per species, for a total of 1,250 leaves per plant species).**

#Step 5
# One leaf per image (Black and white, 50 images per individual plant)

1. **Return to the images saved in step 4.5, and place all 50 leaves in black and white color**
   Use the images that have already been processed individually → return to image 1 (plantname.1.color.png) → right-click → colors → Threshold → click on spreadview and drag the line to check the herbivorous areas and leaf limits → if necessary, increase or decrease the threshold. Repeat this process for the 50 leaves.

2. **Exporting image**

   Export the black and white image by right-clicking on"File", then "Export as..." (shortcut CONTROL + SHIFT + E) and change image name following the pattern:

   plantname.leafnumber. B/W.png

   EX: Bforticata.1B/W.png

   Create a new folder named **plantname.leaves.BW** by clicking "Create Folder", if it does not already exist, choose the folder, click "Export" and leave only "Save resolution" and "Save color profile" checked in the settings box. Click export and check whether the file was saved correctly.

3. **Repeat the above process for all leaves (total = 50 leaves per individual plant, 5 replicates per species).**

IMPORTANT NOTES

Do not change the original size of the images.

Use other leaves of the same species as a reference for drawing the boundaries of leaves with herbivory on the margins.

If there is anything unwanted in the photo (e.g. dirt), change the color of the pencil to white, increase the thickness if necessary and use it as an eraser (the white color is not read by EBImage on R).

USEFUL KEYBOARD SHORTCUTS

- E = Elliptical selection tool
- R = Rectangular selection tool
- F = Simple selection tool
- N = Pencil
- CONTROL + MOUSE WHEEL = zoom in and zoom out
- CONTROL + Z = Undo
- CONTROL + O = open file
- CONTROL + X = Record
- CONTROL + V = Paste
- CONTROL + N = New work tab
- CONTROL + A = Select all (a selection tool must be selected)
- CONTROL + SHIFT + E = export as

***You can create a new keyboard shortcut to open the threshold tool and make the image black and white faster:**
Menu > Edit > Keyboard shortcuts, a window will open with all the program's shortcut keys > type Threshold > click on the word disabled > press the keys you want to use for this function (e.g. press Control + T). So, every time you need to use the threshold, just press control+T.

R code for herbivory analyses on EBImage is available on Figshare in Cornelissen (2024) at https://doi.org/10.6084/m9.figshare.27123372.v1.

References

Cornelissen, T. 2024. Files associated to The Handbook of Herbivory quantification. Figshare. [Dataset.] https://doi.org/10.6084/m9.figshare.27123372.v1

Mendes, G.M., Silveira, F.A.O., Oliveira, C., Dáttilo, W., Guevara, R., Ruiz-Guerra, B., et al. 2021. How much leaf area do insects eat? A data set of insect herbivory sampled globally with a standardized protocol. Ecology 102:3301.

Pérez-Harguindeguy, N., Díaz, S., Garnier, E., Lavorel, S., Poorter, H., Jaureguiberry, P., Bret-Harte, M.S., Cornwell, W.K., et al. 2013. New Handbook for Standardised Measurement of Plant Functional Traits Worldwide. Australian Journal of Botany 61: 167-234. https://doi.org/10.1071/BT12225

Section S2

In compliance to ESA Statistical Analysis guidelines to statistical reporting, we provide complete statistical reporting throughout the manuscript and additional information is placed in section 2 of Appendix S1. Information shown here is linked to each one of the figures included in the main manuscript or statistical analyses, when aplicable.

Table S1. Herbivory level on 30 leaves of 24 plant species as a function of the method used to estimate natural herbivory (Figure 3A in main manuscript)

Pairwise Comparisons####

Table Contrast	Estimate (log scale)	SE	df	t.ratio	*p*-value	Significance
Visual_method - ImageJ	2.41	0.51	65.2	4.71	< 0.0001	a vs. b
Visual_method - EBImage	2.39	0.51	65.2	4.67	< 0.0001	a vs. b
Visual_method - HerbiEstim	1.02	0.52	65.2	1.96	0.20	a vs. c
ImageJ - EBImage	-0.02	0.51	65.2	-0.04	0.99	b vs. b
ImageJ - HerbiEstim	-1.39	0.52	65.2	-2.67	0.04	b vs. c
EBImage - HerbiEstim	-1.37	0.52	65.2	-2.63	0.05	b vs. c

Table S2. Herbivory level of 100 leaves of 20 plant species with artificial herbivory as a function of the method used to estimate herbivory (Figure 3B in main manuscript).

###Pairwise Comparisons###

Contrast	Estimate (log scale)	SE	df	t.ratio	*p*-value	Significance
Visual_estimates - ImageJ	5.12	0.29	297	17.6	< 0.0001	a vs. b
Visual_estimates - EBImage	5.08	0.29	297	17.4	< 0.0001	a vs. b
Visual_estimates - HerbiEstim	4.95	0.29	297	16.9	< 0.0001	a vs. b
ImageJ - EBImage	-0.04	0.29	297	-0.14	0.99	b vs. b
ImageJ - HerbiEstim	-0.17	0.29	297	-0.58	0.93	b vs. b
EBImage - HerbiEstim	-0.13	0.29	297	-0.44	0.96	b vs. b

Delta herbivory before and after training in Zax platform (Figure 4B in main manuscript).

- **Paired t-test (one-tailed as training is expected to reduce estimation error)**
- **Exact P value: 0.0000135; P reported in manuscript as P<0.0001**

Difference between visually estimated herbivory versus those measured through image analysis tools as a function of herbivory levels in different groups of leaves. (Figure 5 in main manuscript).

F-statistic ($F_{2,27}$) = 38.476

Exact p-value = 1.64 × 10^{-8} (not rounded)

P reported in manuscript as P<0.0001

Table S3 - Comparisons of delta herbivory across leaf damage categories (see text for category description)

**Comparison**	**Mean Difference**	**Tukey HSD q-value**	**p-value**	**95% CI**	**Significance**
Low vs. Intermediate	-5.22	8.21	< 0.001	[-7.12, -3.32]	***
Low vs. High	-11.01	17.29	< 0.001	[-12.91, -9.11]	***
Intermediate vs. High	-5.79	9.08	< 0.001	[-7.69, -3.89]	***

P **< 0.001** (all comparisons are significant).

CI = Confidence Interval.

Table S4. Post-hoc comparisons of delta herbivory across leaf damage categories (see text for category description)

**Categories Compared**	**Mean Difference**	**p-value**
Low vs. Intermediate	-5.22	< 0.001
Low vs. High	-11.01	< 0.001
Intermediate vs. High	-5.79	< 0.001

Table S5 – Herbivory level of 64 plant species as a function of method used to quantify leaf damage.

Model:

Herbivory ~ Method + (1 | Plant_species)

Output:

Effect	Wald χ^2	df	p-value
Method	15.01	2	0.00055

Table S6 - Pairwise Comparisons Table

**Comparison**	**Estimate**	**SE**	**z-value**	**p-value**	**Significance**
**Visual vs. ImageJ**	+5.21	1.12	4.65	**< 0.001**	***
**Visual vs. EBImage**	+5.03	1.12	4.49	**< 0.001**	***
**ImageJ vs. EBImage**	-0.18	0.98	-0.18	0.982	n.s.

***P **< 0.001**; n.s. = not significant.

Visual estimates are significantly higher than ImageJ/EBImage (no difference between the latter two, P=0.982)

Table S7**.** Post-hoc comparisons of herbivory quantification methods.

**Comparison**	**Mean Difference**	**p-value**
Visual vs. ImageJ	+5.21%	< 0.001
Visual vs. EBImage	+5.03%	< 0.001
ImageJ vs. EBImage	-0.18%	0.982

Table S8 – Time spent, in minutes, to estimate herbivory as a function of the method used.

Model:

Time ~ Method + (1 | Plant_Species)

Output:

Effect	Wald χ^2	df	p-value
**Method**	**106.478**	**2**	**5.51×10^{-24}**

Highly significant effect of method on time spent on herbivory measurements (χ^2 = 106.478, P = 5.51×10^{-24}) (P value was rounded in manuscript to P<0.0001)

Table S9 - Pairwise Comparisons Table:

Comparison	Mean Difference (min)	SE	z-value	p-value	Significance
**Visual vs. ImageJ**	-37.23	3.12	-11.93	**< 0.001**	***
**Visual vs. EBImage**	-50.81	3.12	-16.28	**< 0.001**	***
**ImageJ vs. EBImage**	-13.58	3.12	-4.35	**< 0.001**	***

*****P < 0.001** (all methods differ significantly).

Table S10. Post-hoc comparisons of time (in minutes) across methods.

**Comparison**	**Mean Difference**	**p-value**
Visual vs. ImageJ	-37.23 min	< 0.001
Visual vs. EBImage	-50.81 min	< 0.001
ImageJ vs. EBImage	-13.58 min	< 0.001
